# Optimising the glaucoma signal/noise ratio by mapping changes in spatial summation with area-modulated perimetric stimuli

**DOI:** 10.1038/s41598-018-20480-4

**Published:** 2018-02-01

**Authors:** Lindsay Rountree, Pádraig J. Mulholland, Roger S. Anderson, David F. Garway-Heath, James E. Morgan, Tony Redmond

**Affiliations:** 1 School of Optometry and Vision Sciences, Cardiff University, Cardiff, United Kingdom; 2 Optometry and Vision Science Research Group, School of Biomedical Sciences, Ulster University, Coleraine, N. Ireland United Kingdom; 3 NIHR Biomedical Research Centre, Moorfields Eye Hospital NHS Foundation Trust and UCL Institute of Ophthalmology, London, United Kingdom

## Abstract

Identification of glaucomatous damage and progression by perimetry are limited by measurement and response variability. This study tested the hypothesis that the glaucoma damage signal/noise ratio is greater with stimuli varying in area, either solely, or simultaneously with contrast, than with conventional stimuli varying in contrast only (Goldmann III, GIII). Thirty glaucoma patients and 20 age-similar healthy controls were tested with the Method of Constant Stimuli (MOCS). One stimulus modulated in area (A), one modulated in contrast within Ricco’s area (C_R_), one modulated in both area and contrast simultaneously (AC), and the reference stimulus was a GIII, modulating in contrast. Stimuli were presented on a common platform with a common scale (energy). A three-stage protocol minimised artefactual MOCS slope bias that can occur due to differences in psychometric function sampling between conditions. Threshold difference from age-matched normal (total deviation), response variability, and signal/noise ratio were compared between stimuli. Total deviation was greater with, and response variability less dependent on defect depth with A, AC, and C_R_ stimuli, compared with GIII. Both A and AC stimuli showed a significantly greater signal/noise ratio than the GIII, indicating that area-modulated stimuli offer benefits over the GIII for identifying early glaucoma and measuring progression.

## Introduction

Standard Automated Perimetry (SAP) is regarded as the current clinical standard for identifying glaucomatous visual field damage and change over time^1^. However, it has three cardinal limitations. First, SAP has poor sensitivity to early disease^2^, and although test-retest variability is lowest in early disease and in healthy individuals, it is unacceptably high for the identification of subtle damage^3,4^. Second, the greater variability in visual field locations with moderate damage (which increases with depth of defect), greatly inhibits the timely identification of change in those with established glaucoma^4–6^. Third, the test has a limited useable dynamic range, with test-retest variability spanning almost its entire range in individuals with advanced damage^4,7,8^, such that the measurement of remaining vision is difficult.

Several studies have attempted to address the limitations of SAP by investigating the utility of alternative stimuli and comparing it to that of the clinical standard (Goldmann III). It has been suggested that employment of some alternative stimuli (e.g. the larger Goldmann V stimulus, area: 2.3 deg^2^) could enable measurement of a larger range of damage, with an accompanying reduction in test-retest variability^7,9^. This addresses the ‘noise’ component of the signal-to-noise ratio, but ascertaining whether such stimuli allow the test to maintain the same sensitivity to early damage (‘signal’) is not straightforward. In the absence of a clear rationale for using alternative stimuli, in terms of physiology, beyond reports that they may offer lower measurement variability, it is premature to confirm their superior utility, or otherwise, in clinical testing. Furthermore, a comparison of the utility of different stimuli on existing clinical instruments is not straightforward, particularly if it is not possible to precisely control their parameters, and without a precise knowledge of the workings of the thresholding algorithm employed. A comparison of stimuli with a non-clinical technique such as the Method of Constant Stimuli (MOCS) is also difficult if one is restricted to using the stimulus step size and scale provided on the clinical instrument. This becomes even more challenging if comparing stimuli between different instruments. Such a restriction could well affect the resolution and accuracy with which the psychometric functions can be sampled for different stimuli, thereby increasing the risk of slope bias^10–12^. A full understanding of the diagnostic benefits of using alternative stimuli, requires the removal or minimisation of confounding factors that are unrelated to the stimulus configuration, such as the thresholding algorithm or unequal psychometric function sampling between stimuli.

**Figure 1:**
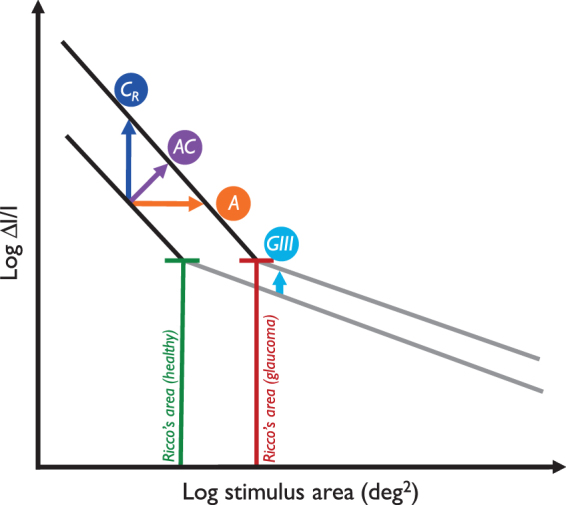
Schematic illustrating the change in spatial summation found by Redmond *et al*.^31^ GIII: Goldmann III stimulus, currently used in SAP. The distance between normal and glaucoma spatial summation curves for a stimulus of fixed area, varying in contrast is small in this region. ‘A’, ‘AC’ and ‘C_R_’ indicate an area-modulated, area-contrast modulated, and contrast modulated (within R_A_) stimulus, where the distance between the spatial summation curves is greater.

The optimisation of stimulus parameters for use in SAP to maximise the signal to noise ratio (SNR), should be based on the underlying physiological mechanisms being measured. Spatial summation describes the way in which the visual system integrates light energy across the area of a stimulus. Ricco’s law states that, for a range of small stimuli, within a critical area (Ricco’s area, R_A_), the intensity of the stimulus at threshold is inversely proportional to its area (threshold x area = constant)^13^. This is referred to as ‘complete spatial summation’. Beyond R_A_, spatial summation is incomplete. R_A_ is not a constant value, and has been found to vary with visual field eccentricity^14–17^, retinal illuminance^18–20^, and stimulus duration^16,21^. Traditionally, R_A_ was thought to have a physiological basis at the retinal level^18,22,23^, however increasing evidence indicates that it is likely a perceptual result of spatial filtering at multiple hierarchies of visual processing, in the retina and visual cortex^24–29^; i.e. the ‘perceptive field’^26,30^. An enlarged R_A_ has been found in patients with primary open angle glaucoma, and differential amounts of sensitivity loss to a range of stimulus sizes can be mapped to a lateral shift in the spatial summation function^31^. The finding has important implications, not only for a better understanding of the pathophysiological changes that occur in glaucoma, but also for the development of methods to identify early subtle damage^26,31^. Pan & Swanson have shown that, rather than probability summation across retinal ganglion cells (RGCs), it is spatial filtering by multiple cortical mechanisms that accounts for perimetric spatial summation^29^. Although glaucoma is characterised by RGC death, it is perhaps unsurprising then, that it is difficult to reconcile perimetric sensitivity and retinal structure, without consideration being given to spatial summation. Given the dependence of the relationship between visual field sensitivity (with conventional stimuli) and underlying RGC density on the relative size of the stimulus and local R_A_^32^, in addition to the variation in R_A_ with visual field eccentricity^16,33,34^, and known changes in R_A_ in glaucoma^31,35^, it makes little sense to continue using an arbitrary fixed-area stimulus to probe the visual field. Rather, a stimulus should be selected in a way that supports meaningful measurements of changes in spatial summation in glaucoma. Anderson^26^ proposed that if early glaucomatous loss were associated with a change in spatial summation in glaucoma, greater attention should be given to the area of the stimulus relative to R_A_; specifically, scaling the stimulus to the local R_A_ in healthy individuals. Given that the intensity threshold at R_A_ is largely constant irrespective of visual field locus^16^, deviations from normal could be measured and compared on an equal par between locations. Redmond *et al*.^31^, having found an enlarged R_A_ in glaucoma, proposed a test paradigm whereby a relative shift in the spatial summation function in glaucoma might be better identified by varying stimulus area during the test, either instead of, or simultaneously with contrast (see Fig. 1 for an illustration of this hypothesis). It is also expected that a stimulus varying in area during the test would enable a greater range of disease to be measured than is the case with current stimuli.

In this study, we test the hypothesis of Redmond *et al*.^31^, as illustrated in Fig. 1, that a stimulus varying in area alone (A), or simultaneously with stimulus contrast (AC), will enable a greater disease signal by directly measuring a shift in an individual’s spatial summation function. In addition, it is hypothesised that the use of such a stimulus, varying in area rather than contrast-only (i.e. one expected to have greater robustness to age-related ocular media changes), will have reduced response variability compared to that found with conventional stimuli. Here, we compare the disease signal, response variability, and SNR for four different stimulus forms, as illustrated in Fig. 1, varying in area, contrast (two stimuli of different, but fixed area), and both area and contrast simultaneously.

## Methods

In this cross-sectional study, psychometric functions were measured with four different stimulus forms (two varying in contrast only, one varying in area only, and one varying proportionally in contrast and area simultaneously) in patients with glaucoma and age-similar controls. Disease signal (deviation in energy threshold from that of age-matched normal), noise (response variability) and SNR were determined and compared between stimulus forms.

### Participants

Thirty patients with glaucoma (median [IQR] age: 70.5 years [66.5, 74.7]; median [IQR] MD: −4.04 dB [−9.30, −2.78]) and 20 age-similar healthy participants (median [IQR] age: 67.3 years [62.0, 75.1]; median [IQR] MD: + 0.33 dB [−0.40, +0.77]) were recruited and tested. All of the glaucoma patients had received a diagnosis of primary open angle glaucoma, 18 with high tension and 12 with normal tension glaucoma, by the hospital eye service. Glaucoma severity varied from minimal field loss (‘within normal limits’ on the Glaucoma Hemifield Test) to ‘advanced’ field loss (categorised with the Hodapp-Parrish-Anderson glaucoma grading scale)^36^, with the SITA Standard 24-2 program on the HFA II. All healthy participants had a full visual field (‘within normal limits’ on the Glaucoma Hemifield Test).

SAP (HFA II, SITA Standard 24-2 program) was performed twice in the test eye prior to any experimental tests, or once if participants had undertaken one of these tests within the past six months as part of their routine clinical care; this ensured that participants had adequate perimetric experience before undertaking experimental tests. False positive rates were <15% for all participants.

Participants did not have any other systemic/ocular disease and/or medication known to affect visual performance (e.g. diabetes, thyroid disease, age-related macular degeneration, hydroxychloroquine medication); ocular health was confirmed by slit lamp biomicroscopy. One glaucoma patient had previously undergone trabeculectomy surgery in the test eye seven years prior to the study, but otherwise no participants had undergone ocular surgery, with the exception of cataract removal. All participants had an intraocular pressure (IOP) <21 mmHg, measured with Goldmann Applanation Tonometry. Healthy participants did not have any first-degree relatives with glaucoma, and did not have any history of elevated IOP.

All participants had a best-corrected visual acuity of ≥6/9 in the absence of significant corneal or media opacities (≤NO3, NC3, C3 and/or P3, Lens Opacities Classification System III)^37^, spherical refractive error between + 6.00 DS and −6.50 DS, and astigmatism <3.50 DC in the test eye, as determined by a full refraction conducted before the commencement of any experimental tests. All experimental tests were conducted with natural pupils, and with participants wearing full refractive error correction for a working distance of 30 cm.

One eye of each participant was tested. The test eye was selected as the eye that best met the inclusion/exclusion criteria, or was selected at random if both eyes were equally suitable. Participants completed each of the four experimental tests on four separate visits (in randomised order) within a four-month period.

Ethical approval for the study was given by the East of Scotland Research Ethics Committee (NHS Scotland). The research adhered to the tenets of the Declaration of Helsinki. Written, informed consent was obtained from all participants prior to inclusion.

### Apparatus and Stimuli

All stimuli were displayed on a gamma-corrected, 25” organic light-emitting diode (OLED) display (Sony PVM-A250 Trimaster El, resolution 1920 × 1080 pixels, frame rate 60 Hz, refresh rate 120 Hz), driven by a ViSaGe MKII Stimulus Generator (Cambridge Research Systems, Rochester, UK). Experiments were programmed in MATLAB (2014b, The MathWorks, Inc., Natick, MA), using the ‘CRS’ toolbox. A uniform background luminance of 10 cd/m² was used.

In order to directly compare the performance of each stimulus form, all stimulus steps were converted to a common scale with identical units (energy = increment luminance x duration x area, units: cd/m^2^.s.deg^2^). Step sizes were approximately equal, in terms of log energy, across stimulus forms, and with a common reference value.

Four visual field locations were tested, 9.9° from fixation along the 45°, 135°, 225° and 315° meridians. The four different stimulus forms compared in this study, varied in the following parameters during experiments:

#### Contrast only (within Ricco’s area, “C_R_”)

A stimulus of fixed area (−1.93 log deg²), varying in contrast only. This stimulus area was determined as the 0.1 percentile of R_A_ values in healthy observers from the study of Redmond *et al*.^31^. To aid appreciation for the size of this stimulus, it is larger than a Goldmann I stimulus (−2.04 log deg²) and smaller than a Goldmann II stimulus (−1.44 log deg²) found on some commercially available perimeters. Possible log stimulus contrast ranged from −1.66 (log ∆I/I, increment luminance 0.22 cd/m^2^) to 1.30 (log ∆I/I, increment luminance 198.85 cd/m^2^).

#### Area only (“A”)

A stimulus of fixed contrast (log ∆I/I: −0.30, increment luminance 4.98 cd/m²), varying in area only, from a R_A_-scaled starting area. This contrast was determined as the predicted threshold for a stimulus area of −1.73 log deg^2^ (the 2.5^th^ percentile of R_A_ values in healthy observers from Redmond *et al*.^31^). Possible stimulus areas ranged from −2.52 log deg² to 2.16 log deg². This maximum was chosen to avoid stimuli crossing the horizontal or vertical midlines, or overlapping with other test locations.

#### Area and contrast simultaneously (“AC”)

A stimulus varying simultaneously and proportionally in area and contrast, such that the slope of modulation was +1 in contrast/area space (Fig. 1). The smallest stimulus had an area of −2.52 log deg², with a contrast of −0.98 log ∆I/I (increment luminance 1.05 cd/m²), and the largest stimulus had an area of −0.11 log deg², with a contrast of 1.28 log ∆I/I (increment luminance 188.60 cd/m²). As the stimulus modulation came from both area and contrast, the maximum area for this stimulus was limited by the luminance capabilities of the display.

#### Contrast-only (Goldmann III-equivalent stimulus, “GIII”, reference stimulus)

A stimulus of fixed area (−0.95 log deg², Goldmann III-equivalent stimulus), varying in contrast; this employed the same contrast scale as the C_R_ stimulus. As the Goldmann III stimulus is the conventional stimulus employed in SAP, this was included here as a reference stimulus.

In order to directly investigate the effect of stimulus configuration on disease signal, response variability and SNR, it was necessary to control, as much as possible, for any artefactual bias that could arise from the method used to determine these parameters. For example, we wished to control for a situation in which a test with one stimulus form could contain more suprathreshold presentations than one with another stimulus form, resulting in an artefactual steepening/flattening of the psychometric function. Thus, stimulus visibility was equated across all stimuli in each observer, as described below.

### Psychophysical Procedure

**Figure 2:**
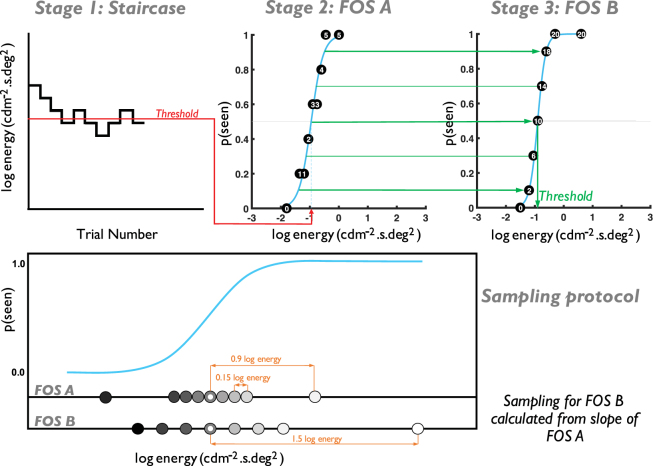
Top: Schematic of the 3-stage process for finding threshold and response variability for each stimulus form. Stage one: short 1:1 staircase procedure. Stage two: short MOCS (5 presentations per level), using the threshold from stage one to select presented energy values. Stage three: standard MOCS (20 presentations per level), using FOS slope from stage two to inform the presented energy values (see Methods for a full description). Bottom: Illustration of the sampling protocol for FOS experiments.

Psychometric functions were measured at each location with each of the stimulus forms (separately) with a MOCS procedure. In an attempt to maximise efficiency, minimise slope bias, and equate stimulus visibility across all conditions, a protocol was adopted whereby the psychometric function was densely sampled around the expected p(seen) = 0.5 region (50% seen, energy threshold), guided by the work of Hill^10^, with sufficiently supra- and sub-threshold stimuli presented on the expected position of the asymptotes (Fig. 2). To do this, frequency-of-seeing (FOS) curves were constructed using a three-stage approach, as follows (see Fig. 2 for an illustrated guide).

#### Stage One – Staircase procedure

To plan a sampling protocol for the MOCS (stages two and three, below) it was necessary to perform a short 1:1 staircase procedure to determine an approximate energy threshold for each of the four visual field locations (interleaved). The staircase terminated after six reversals. Stimulus energy increased/decreased by 0.5 log energy following the first reversal, with proportionally smaller step sizes following each subsequent reversal. Energy was modulated in 0.05 log unit steps (the minimum possible step size) following the fourth and fifth reversals. The threshold at each location was taken as the mean of the final four reversals. The staircase procedure was performed twice, allowing participants the opportunity to become familiar with the stimulus form. Energy threshold values from the second test were then used in stage two.

#### Stage Two – FOS A

The purpose of this stage was to determine an approximate FOS curve position and slope, in order to optimise sampling of the curve in stage three. This stage consisted of a short MOCS procedure, using nine energy levels, each presented five times at the four visual field locations (180 presentations). The nine energy levels were the energy threshold from stage one, three above and three below this initial energy threshold level, each separated by 0.15 log energy, and two further values, 0.9 log energy above and below the initial energy threshold level.

Presentations were randomised in terms of energy level and test location. A rest break was taken halfway through the test. A FOS curve was constructed from the results for each of the four visual field locations, and fitted with a psychometric function. Energy values at p(seen) = 0.1, 0.3, 0.5, 0.7, and 0.9 were estimated from the curve and used to sample the psychometric function in stage three.

#### Stage Three – FOS B

In this stage, participants were presented with 20 repetitions of eight energy levels at each of the four visual field locations (640 presentations). At each location, five of the energy levels were determined from the FOS curve for the same location in stage two (values for p(seen, 0.1, 0.3, 0.5, 0.7, and 0.9)). Three additional energy levels were presented; two levels were p(seen, 0.5) ± 2 standard deviations (SD) from the psychometric function (IQR/1.349) in stage two, and one additional level high above threshold (p(seen, 0.5) + 1.5 log energy), to ensure that a greater number of stimuli were supra-threshold than sub-threshold and thus aid observer attention. The energy levels at all locations were randomly presented. A rest break was taken at every quarter (after 160 presentations). FOS data were fitted with a logistic psychometric function, with guess and lapse rates allowed to vary between 0 and 0.1 (0–10%). The energy threshold was established as the energy value at p(seen) = 0.5. Response variability was taken as the SD of the psychometric function (IQR/1.349). These values were used in subsequent analysis of signal, noise, and SNR.

During all tests, participants were instructed to fixate a central cross and respond to any perceived stimulus by pressing a button on a response pad (Cedrus RB-530). Stimulus duration was fixed at 200 ms for all experiments.

### Statistical Analysis

Fitting of psychometric functions, and analysis of FOS data were performed in MATLAB (version R2015b; The MathWorks Inc., Natick, MA, USA), using the ‘Palamedes’ toolbox^38^. Analyses described from this point were conducted on those FOS data collected in stage three.

#### Total deviation (TD)

To examine differences in disease signal between stimulus forms, energy thresholds for healthy participants were pooled across the four locations, plotted against age for each of the four stimulus forms, and fitted with a mixed model linear regression. TD was then calculated for each location in glaucoma patients as the difference between measured threshold and that of an age-matched normal, estimated from the linear regression model for that stimulus form. TD values for the GIII stimulus were pooled across all locations in all glaucoma patients, and divided into three TD strata: lower (between the 99^th^ and 66^th^ percentiles, equivalent to a localised sensitivity of >28.4 dB with HFAII), middle (between the 66^th^ and 33^rd^ percentiles, equivalent to a localised sensitivity between 24.6 and 28.4 dB) and upper (within the 33^rd^ percentile, equivalent to a localised sensitivity <24.6 dB). TD values for A, AC and C_R_ stimuli were plotted against those measured with the GIII stimulus, and the residuals for each stimulus from a line of equation x = y (i.e. the GIII stimulus plotted against itself) were examined to determine whether TD values were higher or lower, overall, than those with the GIII stimulus.

#### Response variability

To test the hypothesis that a stimulus varying in area has lower response variability compared to conventional stimuli, response variability was compared between all stimuli at each location in the lower stratum with a Friedman test. In addition, to determine the association between response variability and disease severity for each stimulus form, total least squares linear regression was performed on these data at each location. In our analysis, a psychometric function slope of zero (i.e. a vertical slope) represented the ideal observer, with larger values representing flatter slopes. Therefore, in the total least squares analysis, steeper regression line slopes indicate more marked dependence of response variability on TD, while a regression slope of zero indicates that response variability is independent of TD.

#### Signal/Noise Ratio (SNR)

As neither disease signal, nor response variability alone can fully inform the utility of one stimulus over another, SNR (TD/response variability) was compared between stimulus forms, and across the three disease strata. A linear mixed effects model analysis of the relationship between SNR and stimulus form was performed on SNR data pooled from all four test locations. Stimulus form and stratum (without an interaction term) were entered as fixed effects. Intercepts for subjects and test locations, as well as by-subject random slopes for the effect of stimulus form were entered as random effects. There were no obvious deviations from normality, nor heteroskedasticity. Likelihood ratio tests of the model including the effect in question, against the same model excluding the effect, were used to determine p-values.

In all statistical analyses, a Holm-Bonferroni post-hoc correction was applied where there were multiple tests of the same hypothesis. Statistical analysis was performed with the open source statistical environment R^39^, and the lme4 package^40^, where applicable.

The datasets generated and analysed during the current study are available from the corresponding author on reasonable request.

## Results

**Figure 3:**
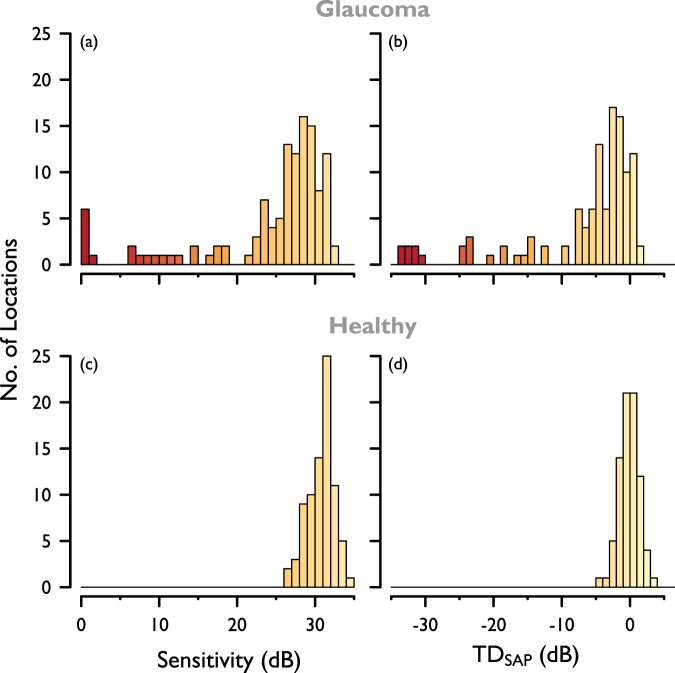
Baseline SAP visual field sensitivity, interpolated for the four visual field locations used in this study. (**a**) SAP sensitivity and (**b**) TD_SAP_ for participants with glaucoma; (**c**) SAP sensitivity and (**d**) TD_SAP_ for healthy participants.

As a clinical indicator of the severity of local damage examined in this study, sensitivity and Total Deviation (TD_SAP_) were interpolated for the four visual field test locations from the final preliminary SAP test (HFA II, SITA Standard 24-2), and plotted in Fig. 3.

In stage three, mean guess rate was 0.01 (1%) for both healthy and glaucoma participants, and mean lapse rate was 0.02 (2%) and 0.04 (4%) for healthy and glaucoma participants respectively.

### Total deviation

Of the 120 test locations across the glaucoma cohort (30 patients, four visual field locations), energy thresholds, and therefore TD, could not be established at two locations for the A stimulus, 15 for the AC stimulus, 42 for the C_R_ stimulus, and 21 for the GIII stimulus. This was due to incomplete FOS curves, in that a p(seen) = 0.5 (50% seen) value could not be reliably determined owing to the different dynamic ranges of the stimulus forms with the apparatus used. There were no incomplete FOS curves with any of the four stimulus forms in the healthy cohort. To compare stimulus forms directly, independent of dynamic range, a separate analysis was conducted on only those locations in which a TD could be established with all four stimulus forms (‘matched’ data), in addition to analysing all available data (‘complete’ data).

**Figure 4:**
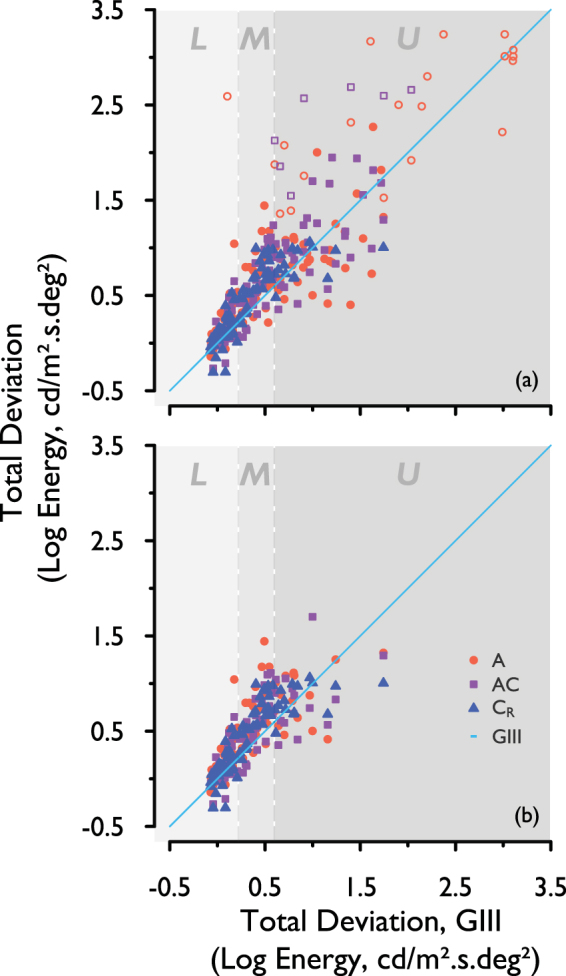
TD for each stimulus form, plotted against TD for the GIII, for (**a**) complete and (**b**) matched data, pooled across the four visual field locations. Light-blue line: TD with the GIII (reference) stimulus. L, M, U: Lower, middle, and upper strata representing three levels of disease severity studied here. Unfilled data points: interpolated SAP sensitivity converted to TD, where TD could not be measured for the GIII with the experimental apparatus.

Figure 4 shows TD values for complete (Fig. 4(a)) and matched (Fig. 4(b)) data for all stimuli in patients with glaucoma. TD for each stimulus form has been plotted against TD for the reference (GIII) stimulus. The blue line indicates TD for the GIII stimulus (i.e. plotted against itself), and as such is used as a reference line. Data points above this reference line indicate a greater TD for that stimulus form than for the GIII stimulus, and those below the reference line indicate a lower TD. ‘L’, ‘M’ and ‘U’ denote the lower, middle and upper strata, according to the TD for the GIII stimulus (as described above). A negative value for TD denotes a lower threshold than that of the age-matched normal threshold. Unfilled data points denote those in which TD could not be established with the GIII stimulus, but could be established with other stimulus forms. These test stimulus TD values were plotted instead against TD calculated from sensitivity measured with the SITA Standard 24-2 program on the HFA II. To explain, sensitivity (dB) values for each of the test locations were interpolated from SAP sensitivity measured with the HFA II grid, and converted from dB values to contrast thresholds. TD was then calculated as the difference between these contrast thresholds and those of age-matched normals, using the normative experimental data in the current study, in the same way as described in the *Methods (Statistical analysis, Total Deviation, TD)* section. TD values for the test stimuli were then plotted against the TD values calculated from SAP sensitivities. The unfilled data points are presented for illustration purposes, and these data were not used in further analysis.

The residuals of the data points in Fig. 4 (i.e. the difference between TD for each of the A, AC and C_R_ stimuli, and TD with GIII) were calculated for both complete and matched data (excluding unfilled data points). These were then averaged, to indicate whether there was an overall larger, or smaller disease signal with each of the three test stimuli compared to that with the GIII and which stimulus form gave the greatest increase. These values are shown in Table 1; average residuals for A, AC and C_R_ were all positive, indicating that overall, TD, and therefore disease signal, with all three test stimulus forms was higher than that with the GIII. This was true for both complete and matched data, with the A stimulus showing the largest signal overall (matched data).

**Table 1:** Mean difference in TD from that with GIII for each of the three stimulus forms. Larger numbers indicate larger overall disease signal than that with the GIII. Unfilled data points in Fig. 4 were not included in the calculation.

Complete data	
	**Mean residuals (TD difference from GIII)**
A	0.08
AC	0.09
C_R_	0.09
**Matched data**	
A	0.14
AC	0.08
C_R_	0.09

### Response variability

Of the 120 test locations across the glaucoma cohort, response variability could not be established in 3 for the A stimulus, 17 for the AC stimulus, 49 for the C_R_ stimulus, and 24 points for the GIII stimulus. As with the energy threshold estimates, this was due to an inability to measure a complete FOS curve at these locations.

**Figure 5:**
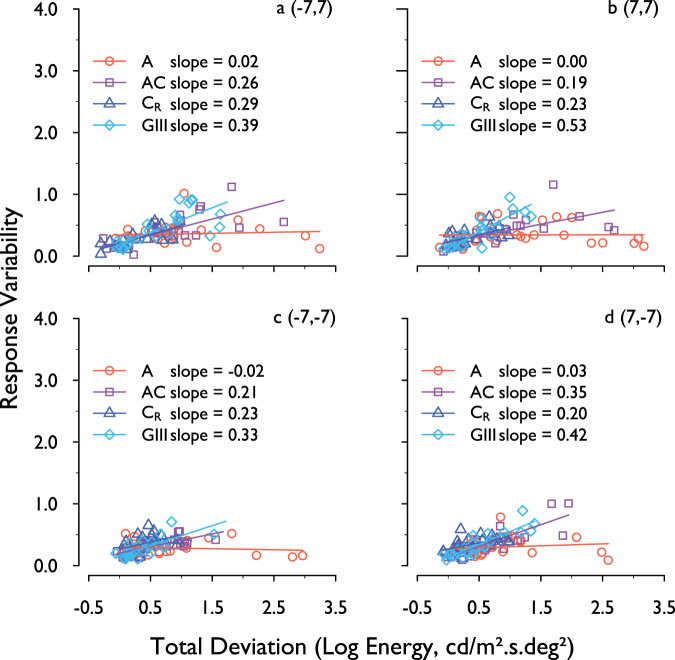
Response variability for each stimulus form, plotted against its own TD for that stimulus form. Data are for each of the four visual field locations (complete data) and are fitted with a total least squares linear regression line.

Figure 5 shows the response variability at all levels of disease severity at all visual field locations for complete data. Response variability for each stimulus form has been plotted against TD for that stimulus form, and a total least squares regression model was fitted to the data. Slope values are shown for each stimulus form.

A Friedman analysis, with post-hoc Wilcoxon signed-rank tests, was performed on the matched data in the lower stratum. Response variability was found to be statistically significantly higher with the A stimulus compared to that with the GIII stimulus at the (−7, −7) location only (p = 0.048, following Holm-Bonferroni correction). No other statistically significant differences were found between any other stimulus forms at any location (all p > 0.05).

Total least squares regression slopes were steepest with the GIII stimulus at all visual field locations, indicating that response variability was most dependent on depth of defect with this stimulus. The shallowest slopes (least dependence on depth of defect) were found with the A stimulus at all locations, followed by C_R_, then AC.

### SNR

**Figure 6:**
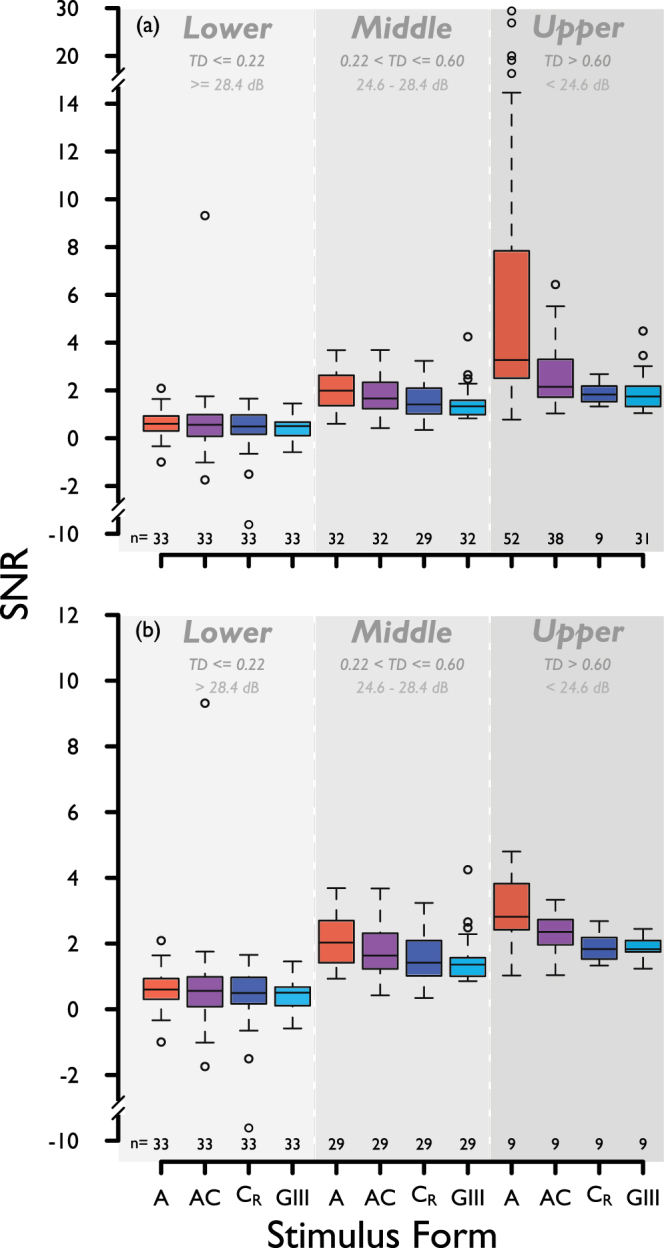
SNR for each stimulus form, for three strata of disease severity, lower, middle and upper percentiles (according to TD with the GIII stimulus, same strata as in Fig. 4. Boundaries between strata indicated in both log energy, and HFA II-equivalent sensitivity), for (**a**) complete and (**b**) matched data, pooled across all four visual field locations. Sample size (n) is given below each box. The y-axis in (**b**) is scaled up for ease of data visualisation.

**Figure 7:**
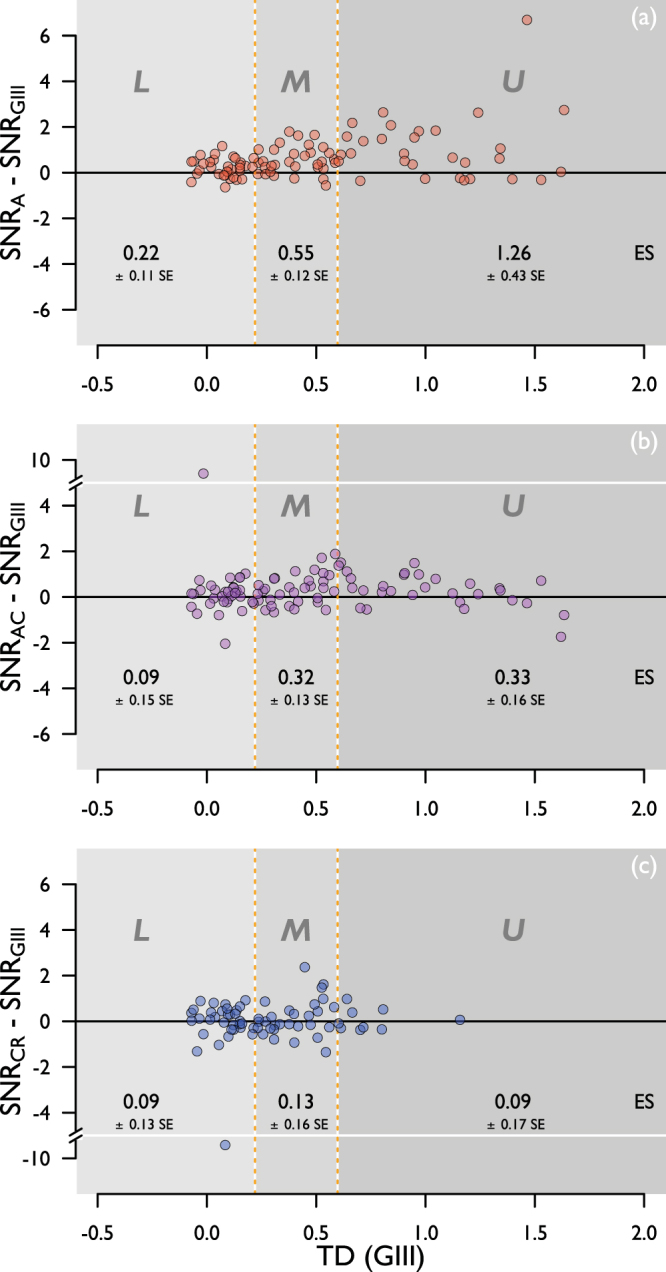
Difference in SNR between GIII and (**a**) A, (**b**) AC, and (**c**) C_R_ stimuli, as a function of defect depth (TD for GIII). L, M, U: Lower, middle, and upper strata representing three levels of disease severity studied here. ES: Effect size in each stratum.

Figure 6 shows SNR for the four stimulus forms, pooled across the four visual field locations, and separated into the same three disease strata according to TD for the GIII stimulus (as described above). SNR is shown for complete (Fig. 6(a)) and matched (Fig. 6(b)) data. One-tailed p-values from the mixed model regression analysis (conducted for matched data only) are displayed in Table 2. Overall, when all three disease strata were considered together, both the A and AC stimuli had a significantly higher SNR when compared with the GIII stimulus, by 0.66 ± 0.15 standard error (SE) (p < 0.001), and by 0.25 ± 0.09 SE (p = 0.008) respectively. SNR for the A stimulus was higher than that for the GIII stimulus in each stratum (p = 0.17, 0.001, and 0.02 in the lower, middle and upper strata respectively). Figure 7 illustrates the difference in SNR between the test stimuli and the GIII, as a function of defect depth (TD for the GIII). It can be seen in Fig. 7(a) that, in each stratum, the majority of data points lie above the line of equality, illustrating a greater SNR for A stimuli than for the GIII at all stages of disease studied here. Effect sizes (difference in SNR from that of GIII, reported by the linear mixed effects analysis) for each stratum are given in Fig. 7. A systematically greater effect size can be seen between lower and upper strata for the A stimulus, while the difference in effect size across strata for the AC and CR stimuli is small. Two data points (those falling outside the split y-axis in Fig. 7, middle and bottom) were considered outliers and were excluded from the linear mixed effects analysis.

**Table 2:** Holm-Bonferroni-corrected one-tailed p-values for overall differences in SNR between stimulus forms, with mixed model regression analysis. *Statistically significant at p < 0.05 level.

Post hoc p-values (all strata)				
	**A**	**AC**	**C**_**R**_	**GIII**
A		0.02*	0.004*	<0.001*
AC			0.27	0.008*
C_R_				0.27
GIII

## Discussion

In order to establish superior utility of a particular stimulus form over another for a given clinical purpose, a greater signal-to-noise ratio (SNR) must be demonstrated with that stimulus. In a trial of a novel stimulus form to be used for discriminating glaucoma from normality, this can be done appropriately by comparing the quotient of the disease signal and response variability with that for the contemporary reference standard. In this study, we have demonstrated a greater overall disease signal, lower dependence of response variability on depth of defect, and greater SNR with stimuli varying in area than for standard Goldmann III stimuli, when compared on a common energy scale and with equivalent visibility. Mindful that an artefactual steepening of the psychometric function could be observed simply by using a psychometric function sampling protocol that enables a greater number of stimulus presentations and larger range of energy levels to be seen above threshold with one stimulus type in comparison to another, stimuli were matched for energy step size and spread of suprathreshold energy levels in the MOCS procedure. We therefore ensured that any differences could reasonably be attributed to stimulus modulation, rather than artefact of experimental design.

In Fig. 4, a greater overall disease signal can be observed with all three test stimuli (A, AC and C_R_) when compared with that for the standard GIII stimulus; the A stimulus showed a larger overall disease signal than the AC and C_R_ stimuli, as denoted by the greater overall TD (Table 1). As this was found with matched data, differing dynamic ranges between stimulus forms do not solely account for the differing disease signals observed here. The overall larger disease signal with the three test stimuli, compared to that for the GIII, is in keeping with the finding of a larger R_A_ in glaucoma patients (displaced spatial summation curve along the area axis), relative to that for age-similar healthy controls^31^, as a difference in threshold to a GIII stimulus represents the distance, on the y-axis, between shallow regions of the spatial summation curves (see Fig. 1) for much of the central visual field. Threshold differences for A, AC, and C_R_ stimuli, on the other hand, represent differences between glaucoma and normal curves in steeper regions of the curve.

It could be assumed that the greater number of locations in which TD was not measurable with the C_R_ stimulus suggests that this stimulus is, in fact, superior at distinguishing between ‘normal’ and ‘glaucoma’ than the other three stimuli. However, the more likely explanation is that this reflects the smaller dynamic range for this stimulus with the hardware used in this study.

We found the greatest increase in response variability with depth of defect for the GIII stimulus (Fig. 5).Response variability was found to be less dependent on disease severity for all three stimulus forms (A, AC and C_R_), with least dependence being observed with the A stimulus. Caution should be exercised at this point, however, as it does not necessarily follow that the stimulus with the lowest or more uniform response variability has the greatest utility for disease detection. To answer this question, one must consider disease signal and response variability together.

Both the A and AC stimuli had a significantly greater SNR than the GIII when all disease strata were considered, but this difference was greatest for the A stimulus. Although notable, the difference in SNR between the A and GIII stimuli is more modest in the lower stratum, likely due to these data representing not only locations with glaucomatous damage, but also those that are relatively healthy. Given that this measure takes account of both disease signal and response variability, and that stimuli are compared on equivalent platforms, scales, and units, more substantial weight can be given to this metric in a comparison of their relative utility. In this study, experiments were performed at locations (±7, ±7); 9.9 deg visual field eccentricity. At this location, and at the background adaptation level employed, the area of the GIII stimulus is close to the normal Ricco’s area. Further investigation in more central locations, using the methodology employed in this study, may help to better understand the utility of area-modulated stimuli in the identification of earlier loss. It is noteworthy that the difference in SNR between the A and GIII stimulus is systematically enlarged across disease strata while the difference in SNR between both the AC and C_R_ stimuli and the GIII remained modest (Fig. 7). Although the utility of area-modulated stimuli for identifying change over time and measuring remaining vision in advanced loss was not formally investigated in this study, this finding raises the possibility that the A stimulus might also outperform the GIII in both regards.

Following reports of substantial RGC loss prior to clinical identification of glaucoma^41–43^, possible vulnerability of RGC subtypes in the condition^44–47^, and concerns about high variability in conventional clinical visual field testing^4,48^, the past few decades have observed a movement to establish test stimuli to identify, with high precision, the subtlest visual field damage. Many studies have previously attempted to compare the diagnostic capabilities of alternative tests with those of SAP. Such a comparison is not straightforward, however, and has been confounded by use of different apparatus, measurement scales, stimulus configurations, adaptation levels, and thresholding algorithms within and between studies. Firm conclusions about the superiority of one stimulus over another cannot be made without control over parameters outside those the manufacturer can provide on a clinical platform. A meaningful comparison of performance between different stimulus configurations can only be made if all other variables, not related to stimulus configuration, are accounted for or minimized. Therefore, caution should be exercised when making conclusions about the utility of one stimulus over another following a comparison on existing clinical platforms.

Redmond *et al*.^31^ previously demonstrated that, in glaucoma, the difference in threshold from normal for a contrast-modulated stimulus close in area to a Goldmann III could be completely mapped to an enlarged R_A_ (their Fig. 5). It therefore follows that stimuli optimised to probe the change in spatial summation function in glaucoma may be more beneficial to identify subtle functional loss in early disease. The results of this study suggest that area-modulated stimuli may offer additional benefits for measuring glaucomatous changes in spatial summation in a clinical setting, in the form of greater disease signal, more uniform response variability with defect depth, and a greater SNR than the conventional fixed-area, contrast-modulated stimuli (Goldmann III) currently employed in SAP. We do not recommend the use of MOCS in a clinical test; this design was chosen in order to ascertain the optimum stimulus modulation paradigm for probing changes in the visual field in glaucoma. Rather, the utility of area-modulated stimuli should now be investigated further by comparison with conventional Goldmann III stimuli on an extended test grid, a common energy scale, common step sizes, and with a common thresholding algorithm, optimised for accuracy and test duration. In this way, the utility of these stimuli for the identification of visual field damage and its progression can be confirmed in the clinical setting.
